# Different Paths, One Goal: Milk Ladders in IgE- and Non-IgE-Mediated Cow’s Milk Protein Allergy—A Narrative Review

**DOI:** 10.3390/nu17243816

**Published:** 2025-12-05

**Authors:** Daria Wiszniewska, Agata Stróżyk, Andrea Horvath, Adam J. Sybilski

**Affiliations:** 1 Department of Paediatrics, Medical University of Warsaw, 02-091 Warsaw, Polandagata.strozyk@wum.edu.pl (A.S.); 2 Clinical Department of Pediatrics and Allergology, National Medical Institute of the Ministry of the Interior and Administration, 02-507 Warsaw, Poland; adam.sybilski@pimmswia.gov.pl; 3 Department of Pediatrics, Centre of Postgraduate Medical Education, 02-507 Warsaw, Poland

**Keywords:** cow’s milk protein allergy, milk ladder, tolerance induction

## Abstract

Cow’s milk protein allergy (CMPA) is one of the most common food allergies in early childhood. Although a strict elimination diet effectively prevents allergic symptoms, it does not promote the development of tolerance to cow’s milk proteins (CMPs). The milk ladder is a stepwise approach to the reintroduction of CMPs, starting with extensively heated forms and gradually progressing to unheated cow’s milk, according to the individual tolerance levels. The rationale for this approach lies in the reduced allergenicity of heated forms, mainly due to the food matrix effect. The milk ladder was originally developed to gradually expand the diet in children with non-IgE-mediated CMPA. However, recent evidence suggests that it can also be applied in IgE-mediated CMPA to accelerate tolerance development. In children with high-risk IgE-mediated CMPA, this approach may be more challenging. Even minor recipe modifications, particularly those affecting composition or heating conditions, can alter the allergenicity of heated foods. Moreover, barriers to reintroducing food allergens, including parental anxiety or the child’s food aversion, may be more pronounced in this group. Although for high-risk patients, reintroduction is usually performed in hospital settings, increasing evidence indicates that, in selected cases, it may also be safely conducted at home. Several emerging strategies, including early low-dose exposure and oral immunotherapy, show promise in further enhancing tolerance development. Despite these challenges, early reintroduction of CMPs appears to play a crucial role in modulating the immune response and promoting tolerance. This review provides the summary of evidence and practical insights into the implementation of the milk ladder in children with IgE-mediated CMPA.

## 1. Introduction

Cow’s milk protein allergy (CMPA) is one of the most common food allergies in early childhood. Reliable epidemiological data regarding CMPA are scarce and primarily refer to the IgE-mediated subtype, with an observed prevalence below 1% of infants [1,2]. However, overdiagnosis is common—based on parent reports, its prevalence may be up to 10% [3]. It typically develops after a child’s exposure to cow’s milk proteins (CMPs) during the introduction of complementary foods or cow’s milk-based infant formulas.

CMPA manifestations involve the gastrointestinal tract, skin, respiratory system, or cardiovascular system. Clinically, CMPA is classified as either IgE-mediated (immediate and potentially severe, with a risk of anaphylaxis) or non-IgE-mediated (delayed and predominantly gastrointestinal) [4,5]. Cow’s milk proteins remain the leading cause of anaphylaxis in children [1,6].

Diagnosis of CMPA relies on a detailed clinical history, supported by skin prick testing and/or specific IgE measurement, while the response to a diagnostic elimination diet followed by an oral food challenge (OFC) remains the gold standard for its definitive confirmation [3,4,5,7].

The natural history of CMPA is generally very favourable, with most children with non-IgE CMPA developing tolerance by 3–5 years of age. In contrast, IgE-mediated CMPA tends to persist longer, often resolving later in childhood [1,8]. Traditional management of CMPA is based on the strict elimination of CMPs from the diet [3,4,7]. However, this approach does not alter the natural course of the allergy. While it is effective in preventing allergic symptoms, long-term elimination results in nutritional deficiencies, feeding difficulties, and psychosocial challenges [9,10]. Consequently, increasing attention has been directed toward proactive strategies that not only avoid allergens but also support the development of immune tolerance.

One promising approach is the milk ladder, a structured and stepwise method for the reintroduction of cow’s milk that begins with extensively heated (baked) forms and gradually progresses to less processed products. This strategy is intended to promote gradual desensitization and the acquisition of tolerance under safe and controlled conditions [11,12,13]. Despite its increasing use in clinical practice, milk ladder protocols remain non-standardized, with substantial variation in the number of steps, food matrices, and progression criteria across existing versions.

The aim of this review is to summarize the current evidence on the use of the milk ladder in children with CMPA, with particular attention to IgE-mediated forms. It provides practical insights into the implementation of the milk ladder, especially in high-risk CMPA phenotypes, and discusses the absence of standardized protocols as well as key clinical and patient-related factors that influence its safety and effectiveness.

## 2. From Baked to Unheated Cow’s Milk—Mechanism Underlying Reduced Allergenicity in the Milk Ladder

**Figure 1:**
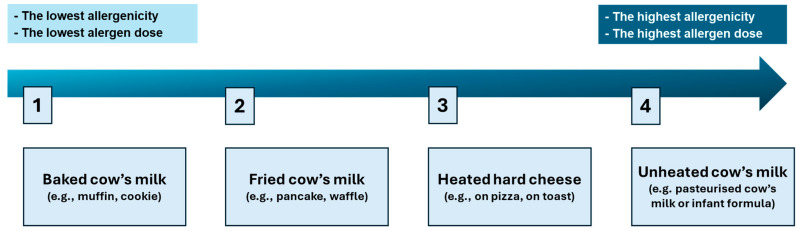
General Concept of the Milk Ladder.

The milk ladder is a structured protocol involving the gradual reintroduction of milk-containing foods according to their increasing allergenicity (Figure 1). It is intended for children with CMPA, mainly those with mild-to-moderate non-IgE-mediated forms, who are on a therapeutic elimination diet, to assess the development of tolerance to CMPs [14].

The milk ladder begins with small amounts of the least allergenic, extensively heat-processed foods (e.g., cookies or muffins containing small amounts of baked cow’s milk proteins) and progresses step by step to larger amounts of less processed forms, ending with an age-appropriate portion of unheated cow’s milk [13,15]. The rationale for its use lies in the reduced allergenicity of baked CMPs when combined with wheat protein, as in cookies and muffins, where extensive heating in the presence of the wheat matrix significantly decreases IgE-binding capacity [12,16]. It is especially important for casein as a linear epitope which remains heat-resistant, in contrast to whey proteins which are easily destroyed by heat (heat-labile) [16].

As a child develops tolerance, subsequent steps introduce less extensively heated foods with higher CMP content, such as pancakes, cheese, yogurt, and finally, unheated pasteurized milk or standard cow’s milk formula. Progression to the next step occurs after a defined period without allergic reactions to the previous food.

The primary goal of the milk ladder in children with CMPA is to promote the development of tolerance through gradual exposure to increasing amounts of less-processed CMPs, potentially accelerating allergy resolution and improving quality of life [13,15]. Achieving tolerance to even baked milk forms may facilitate greater diet diversity, as well as decrease the burden and anxiety for parents related to the risk of accidental allergen exposure [16].

## 3. From Non-IgE to IgE-Mediated Cow’s Milk Allergy—Evolving Concepts of the Milk Ladder

The milk ladder was first introduced in 2013 by the Milk Allergy in Primary Care (MAP) group in the United Kingdom (UK) as a structured method for the gradual home reintroduction of dairy products in children with mild-to-moderate non-IgE-mediated CMPA [17]. It originally included 12 steps based on commonly consumed British foods. In 2017, the international Milk Allergy in Primary Care (iMAP) consortium updated and simplified the MAP Ladder into the six-step iMAP Milk Ladder, and also removed many of the high sugar options to be aligned with the World Health Organization’s recommendations [14].

Children with mild-to-moderate non-IgE-mediated CMPA are generally at very low risk of immediate, potentially life-threatening reactions [8]. These patients mainly present with delayed-onset gastrointestinal or skin symptoms, which makes gradual home reintroduction of CMPs using the milk ladder a safe and practical approach to promoting oral tolerance and expanding the diet.

Encouraged by the promising outcomes seen in low-risk populations with non-IgE-mediated CMPA, researchers have started to explore the use of the milk ladder in higher-risk children with IgE-mediated CMPA.

### 3.1. Efficacy/Effectiveness of Milk Ladder in Children with IgE-Mediated CMPA

While the milk ladder was originally designed for children with low-risk, non-IgE-mediated CMPA, a global survey conducted in 2017 [18] found that up to 60% of healthcare professionals also use it to assess tolerance in IgE-mediated food allergies.

Studies evaluating the efficacy and effectiveness of the milk ladder in children with IgE-mediated CMPA are summarized in Table 1. A 2024 systematic review identified seven studies on this topic, including only one randomized controlled trial (by Esmaeilzadeh et al.) [19]. According to the meta-analysis, 58% of children with CMPA using the milk ladder developed tolerance, whereas allergic reactions occurred in 25% of patients, and severe reactions were reported in only 2% of cases.

#### 3.1.1. Evidence on Efficacy (Randomized Controlled Trials)

To date, three randomized controlled trials have evaluated the effect of introducing baked milk using the milk ladder in children with IgE-mediated CMPA. However, only one trial—by d’Art et al.—did not exclude the high-risk group of children (besides those with a history of anaphylaxis in last 4 weeks) [20].

The earliest trial performed by Esmaeilzadeh et al. [21] demonstrated that in children aged 6 months to 3 years, a stepwise dietary protocol, daily baked milk (muffin) for six months followed by baked cheese (pizza) for another six months, resulted in a higher proportion of children achieving tolerance to unheated milk compared with one year of strict avoidance (88.1% vs. 66.7%, *p* = 0.018, *n* = 84). Additionally, a study by Nowak-Wegrzyn et al. [22], in children aged 4–10 years who tolerated baked milk, found no difference in the proportion of children achieving tolerance to less-processed milk proteins between the 6-month and 12-month escalation schedules (OR = 0.77, 95% CI: 0.31–1.94, *n* = 85). Recently, d’Art et al. [20] also evaluated the efficacy of a single low-dose supervised exposure to whole milk at diagnosis compared with routine care, followed by gradual home introduction using a standardized six-step milk ladder in both groups. Authors found that a greater proportion of children in the low-dose group achieved tolerance to unheated milk compared with routine care (65% vs. 35%, *p* = 0.03, *n* = 60) at 12 months. No severe adverse events were reported in any of these trials [21].

#### 3.1.2. Evidence on Effectiveness (Observational Studies)

Two of three case-control studies did not exclude the high-risk group of children with IgE-mediated CMPA (e.g., uncontrolled asthma, severe atopic dermatitis, history of severe allergic reactions). Trujillo et al. [23] included children with a history of anaphylaxis and reported a high proportion of children with achieved tolerance in the milk ladder group (86.6%) compared with a strict avoidance (86.6 vs. 61%, *n* = 341). Moreover, authors reported a lower incidence of anaphylaxis in the milk ladder group than in the elimination group (3 vs. 34 cases), further supporting the safety and effectiveness of the milk ladder also in the high-risk group of CMPA patients. Similarly, Dunlop et al. [24], who excluded only children with non-IgE-mediated CMPA, in children who failed OFC with BM, observed a higher proportion of children with tolerance to unheated milk in the milk ladder group compared with strict avoidance (30 vs. 10%, respectively).

In a case series by Wiszniewska et al. (2025) and Gallagher et al. (2024), most children with previous anaphylaxis or high sIgE levels (≥5 in children <1 years of age, and ≥15 in children older than 1 year) tolerated at least baked milk products (e.g., cookies, muffins) [15,25]. In both studies, only a few mild, self-limiting reactions were reported, and no cases of anaphylaxis occurred during supervised food challenges.

Although these findings are encouraging, high-quality studies are needed to confirm the efficacy and safety of this approach in high-risk children with IgE-mediated CMPA.

**Table 1:** Summary of studies assessing efficacy/effectiveness of the use of the milk ladder to assess tolerance in children with CMPA.

First Author Year, Country	Design, Sample Size	Child Population	Intervention Group	Control Group	Primary Outcome	Results
*Randomized Controlled Trials*						
Esmaeilzadeh 2018, Iran [21]	RCT *n* = 84	Children aged 6–36 months with IgE-mediated CMPA who passed baked milk OFC.	Consumed BM in the form of a muffin for 6 months and then baked cheese in the form of pizza for another 6 months.	A strict avoidance of cow’s milk for 1 year.	Proportion tolerating baked milk and passing unheated milk OFC (240 mL or at least 8–10 g skim milk) at month 12.	Higher tolerance rate in the intervention group vs. control group (88.1% vs. 66.7%, *p* = 0.018).
Nowak-Wegrzyn 2018, USA [22]	RCT *n* = 85	Children aged 4–10 years with IgE-mediated CMPA who tolerated BM. Only non-reactive children were randomized to the escalation program.	A structured BM escalation program over a 36-month period, with OFCs every 6 months with more allergenic forms of milk (MAFM; muffin → pizza → rice pudding → unheated milk).	A structured BM escalation tried more allergenic (less heat-denatured) forms of milk (MAFM) food challenges with up-dosing every 12 months.	The odds ratio for progression to tolerance of MAFM in the subjects randomized to escalate their dose every 6 months versus those randomized to escalate every 12 months.	No difference in tolerance between 6- and 12-month groups (OR = 0.77, 95% CI, 0.31 to 1.94). Overall, 48% achieved tolerance to unheated milk by month 36.
d’Art 2022, Ireland [20]	RCT *n* = 60	Infants aged <12 months diagnosed with IgE-mediated CMPA.	A single dose of fresh cow’s milk of elicited dose (ED05, 0.5 mg milk protein) followed by the 12-step MAP ML implementation at home.	Routine care, before using the 12-step MAP ML implementation at home.	MAP milk ladder level achieved at 6 months post-randomization. A progressor was defined as reaching step 6 (of 12, lasagne) or higher at 6 months.	Higher proportion of infants reached ≥step 6 of the ML in the intervention group vs. control at 6 months (73% vs. 50%, *p* = 0.048, *n* = 57) and at 12 months (86% vs. 75%). At 12 months, more children completed the ML in the intervention group vs. control (65% vs. 35%, *p* = 0.03).
*Case-control studies*						
Dunlop 2018, USA [24]	Case-control study (with matched retrospective control group) *n* = 206	Patients aged 4 months–20 years (median 6.8 years) with IgE-mediated CMPA who underwent BM OFC between 2009 and 2014 and had ≥24 months of follow-up.	Those who passed the BM OFC were instructed to begin home-based introduction following the four-step ML. Group 1: Passed BM OFC—ML introduction. Group 2: Failed BM OFC—ML introduction.	Group 3: Failed BM OFC—strict avoidance.	Number of children tolerant to BM (muffin), lesser BM (pancake/waffle), baked cheese, and direct milk at final follow-up.	Group 1: 54% tolerated direct milk, 19% avoided milk. Group 2: 29% progressed to direct milk, 38% avoided all milk. Group 3: 10% progressed to direct milk, 85% avoided milk.
Efron 2018, Israel [26]	Case-control study *n* = 110	Children aged 1–4 years with IgE-mediated CMPA, treated between 2011 and 2016, who passed BM OFC.	Structured four-step ML protocol with supervised OFCs and 3-month intervals between consecutive steps.	A strict milk avoidance until 4 years old.	Age at resolution of CMPA (tolerance to 250 mL raw milk) and proportion of children achieving this tolerance.	Median age of CMPA resolution was lower in the intervention vs. control group (36 vs. 96 months, *p* < 0.001). At last follow-up, more children in the intervention group tolerated unheated milk (86% vs. 52%, *p* = 0.003).
Trujillo 2024, Ireland, Spain [23]	Case-control study (analysis of two cohorts) *n* = 341	Children with IgE-mediated CMPA (immediate cutaneous, gastrointestinal, respiratory, or systemic [anaphylaxis] symptoms; SPT ≥ 3 mm or sIgE > 0.35 kU/L), treated between 2011–2020.	Gradual home introduction of BM products following the 12-step IFAN ML, with regular symptom monitoring and allergology follow-up (Irish cohort).	A strict milk avoidance and re-assessment of tolerance development each 6–12 months (Spanish group).	Successful reintroduction of CMPs, defined as daily intake of >150 mL fresh pasteurized cow’s milk (4.5 g milk protein) without symptoms.	Successful milk reintroduction: 86.6% in the ML group (148/171) vs. 61% in the strict avoidance group (122/200); The ML group was 3.67 times more likely to succeed (*p* < 0.001).
*Case-series*						
Kim 2011, USA [13]	Case-series study * n = 148	Children aged 0.5–21 years with IgE-mediated CMPA.	BM-tolerant subjects (*n* = 65) consumed BM products daily; after 6 months were challenged with baked cheese, and baked cheese–tolerant children were subsequently challenged with unheated milk.	BM-reactive subjects (*n* = 23) avoided all milk forms and were re-challenged 6 months after the initial test.	Outcomes of children incorporating baked milk products into their diets.	BM-tolerant: 39 (60%) tolerated unheated milk, 18 (28%) tolerated BM/baked cheese, 8 (12%) continued strict avoidance. BM-reactive: 2 (9%) tolerated unheated milk, 3 (13%) tolerated BM/baked cheese, 78% continued strict avoidance.
Ball 2021, UK [27]	Retrospective case-series *n* = 86	Children aged ≥12 months and <3 years with IgE-mediated CMPA, treated between 2012–2017.	Gradual home introduction of BM products following the four-step ML, with regular symptom monitoring and allergology follow-up every 4–6 months (four visits planned in total).	-	Efficacy and safety of the home-based ML protocol assessed by tolerance acquisition and occurrence of allergic reactions. Tolerance evaluated using a 7-point ML-based scale (0 = no tolerance, 6 = normal diet).	After four reviews, 8 patients still not tolerating most dairy products; 7 lost to follow-up. Allergic symptoms in 81/189 (43%) dietetic reviews, 65 (80%) related to ML.
Gallagher ** 2024, Ireland [15]	Retrospective case-series *n* = 171	Children aged <3 years with IgE-mediated CMPA (symptoms of IgE-mediated allergy, SPT ≥ 3 mm with negative control 0 mm and/or sIgE > 0.35 kUA/L), treated between 2011–2021.	The 12-step ML (modified iMAP ML).	-	Introduction of unrestricted cow’s milk or raw egg, defined as a daily intake of >150 mL cow’s milk (≈4.5 g milk protein) or >30 g raw egg (e.g., meringue, mayonnaise) without symptoms.	Completion of ML: 77.8% of children with anaphylaxis and 88.9% without; OR = 0.438 (95% CI: 0.169–1.133).
Wiszniewska ** 2025, Poland [25]	Retrospective case-series *n* = 15	“High-risk” children with IgE-mediated CMPA (confirmed by positive milk OFC and/or sIgE, and/or SPT), defined as having asthma, anaphylaxis or severe allergic symptoms, sIgE ≥ 15 kUA/L, and/or SPT wheal > 8 mm.	Gradual introduction of BM products using a 5-step ML (modified iMAP). Step 1 (muffin) performed as hospital-based OFC; subsequent steps performed as home reintroduction or additional OFCs, with home introduction safety assessed individually.	-	Number of children with treatment success, defined as negative OFC with raw pasteurized/modified cow’s milk (120–240 mL) or one soft-boiled/lightly scrambled egg (unrestricted intake).	Development of tolerance: 5/15 (33%) achieved tolerance to unheated milk; 11/15 (73%) to BM. No anaphylaxis occurred during hospital-based OFCs.

* Due to the methodological concerns, for the purpose of this review, this study was reported as a case-series including the BM-tolerant and -reactive patients from the same cohort, and excluding the historical control group. ** Wiszniewska et al. and Gallagher et al. [15,25] included patients with cow’s milk and hen’s egg allergies; however, for the purpose of this analysis, only the milk ladder arm was reported. Abbreviations: BM—baked milk, CI—confidence interval, CMPA—cow’s milk protein allergy, IFAN—Irish Food Allergy Network, iMAP—international Milk Allergy in Primary Care, MAP—Milk Allergy in Primary Care, ML—milk ladder, OFC—oral food challenge, OR—odds ratio, SPT—skin prick test, sIgE—specific Immunoglobulin E.

### 3.2. Milk Ladder in the Scientific Organizations Statements

Despite growing clinical interest, evidence supporting the safety and efficacy of the milk ladder in children with CMPA remains limited. As a result, no strong recommendations for or against its use have been established [3,16,28,29,30,31]. Nevertheless, all major scientific societies endorse its use in children with mild-to-moderate non-IgE-mediated CMPA (defined as mild gastrointestinal and/or skin symptoms, e.g., irritability, constipation, mild diarrhea, abdominal discomfort, pruritus, erythema, rash, moderate atopic dermatitis). A summary of official statements on the reintroduction of CMPs in children with CMPA developed by leading organizations is presented in Table 2. However, some concerns have been raised.

According to the European Society for Paediatric Gastroenterology, Hepatology and Nutrition (ESPGHAN), standardization of home-based reintroduction protocols using the milk ladder, adapted to local dietary practices, is needed [3]. Furthermore, the World Allergy Organization (WAO), in its *Diagnosis and Rationale for Action against Cow’s Milk Allergy (DRACMA)* guideline, emphasized the lack of evidence regarding the optimal timing for reintroducing cow’s milk proteins [4].

There is also ongoing debate concerning use of the milk ladder in high-risk patients with CMPA. The GPIFN and MAP (2019) guidelines do not recommend its use in children with IgE-mediated or severe non-IgE-mediated CMPA [30]. In contrast, the recently published European Academy of Allergy and Clinical Immunology (EAACI, 2025) guidelines on IgE-mediated food allergy emphasize the importance of individualized dietary management, including the introduction of baked milk or egg when clinically appropriate [31].

## 4. Milk Ladders—A Need for Standardization

### 4.1. National Milk Ladders Protocols

Since the development of the MAP Milk Ladder in 2013 [17] and its subsequent update by the international iMAP consortium in 2017 [14], numerous national adaptations have been introduced (Table 3). The growing number of milk ladder protocols reflects the need to tailor foods and recipes to local dietary habits, product availability, and cultural preferences [29,30,33,34,35,36,37,38]. Existing milk ladder protocols differ in the number of steps (ranging from 4 to 12), the number of foods per step (from 1 to 6), the types of foods included, the tested doses of cow’s milk proteins, and the overall duration of the reintroduction process. This considerable heterogeneity poses a particular challenge when milk ladders are used to assess tolerance in children with IgE-mediated CMPA [29,30,33,34,35,36,37,38]. Therefore, standardization of milk ladder protocols, especially regarding allergen dose, timing between steps, and total duration of reintroduction, is strongly needed [16].

#### Milk Ladder Duration

The break periods between consecutive milk ladder steps vary from one week to several weeks or months, depending on the protocol and clinical context [15,20,21,27,39,40]. Currently, no optimal duration for each step, interval between steps, or minimum and maximum time required to complete the protocol has been established [4,16,41].

In clinical practice, the duration of each step and the interval between milk ladder stages are usually individualized based on [15,25]:

- History of allergic reaction;
- Child’s age;
- Type of CMPA;
- Severity of symptoms;
- Practical considerations such as parents’ level of anxiety and preferences.

In children with multiple food allergies, the intervals between consecutive milk ladder steps are often shortened for practical reasons, as prolonged reintroduction of CMPs may delay the assessment of tolerance to other food allergens. Despite limited evidence, this approach has not been shown to increase the risk of adverse events when careful monitoring is maintained [15,25].

Currently, no head-to-head studies have compared milk ladders of different durations. However, in children with hen’s egg allergy, one RCT demonstrated a higher rate of tolerance development in the short-arm group compared with the long-arm group (median duration of the egg ladder: 24 vs. 30 months; 80% vs. 69%, *n* = 78) [42]. An ongoing study by Wiszniewska et al. (conducted by our research team) is evaluating tolerance development using four-step and six-step milk ladders with 4-week intervals between steps (16 and 24 weeks in total, respectively) [25]. This study aims to provide further insight into the role of milk ladder duration in tolerance acquisition.

Evidence from RCTs has shown that early introduction of food allergens significantly reduces the risk of developing food allergies [43]. However, available data are insufficient to confirm an association between early introduction of cow’s milk and the risk of CMPA. Although further RCTs are needed to clarify this relationship, experts generally support earlier introduction of food allergens, including cow’s milk.

## 5. Milk Ladder in IgE-Mediated CMPA in Clinical Practice

### 5.1. Patients Selection—For Who?

According to the safety checklist proposed by Chua et al. [44], the reintroduction of CMPs using the milk ladder may be considered in children with IgE-mediated milk who:

- Have not experienced recent severe anaphylaxis upon minimal exposure;
- Are clinically stable (e.g., well-controlled asthma, absence of active atopic dermatitis), without a very high level of specific IgE to CMPs (e.g., casein < 0.54 kU/L);
- Do not have other severe food allergies;
- Have caregivers who are trained to recognize and manage mild allergic reactions and have access to rescue medications.

Children with well-controlled asthma and no history of recent severe reactions may be suitable for home-based milk ladder introduction, whereas those at higher risk should begin the process under medical supervision [15,27].

Emerging evidence suggests that milk ladders may also be considered for use in ‘high-risk’ children, such as those with elevated sIgE levels to CMPs (≥5 in children < 1 years of age, and ≥15 in children older than 1 year) and/or a history of anaphylaxis, provided that close clinical monitoring and thorough parental education are ensured (see Section 3.1.2) [15,23,25].

Moreover, to optimize safety and support the maintenance of acquired tolerance, clinicians should consider the following factors before reintroducing CMPs:

- The child’s willingness to consume foods included in the milk ladder, including acceptance of their taste and texture; regular intake (at least three times per week) of an age-appropriate portion containing a tolerated amount of CMPs is recommended to sustain tolerance once achieved [20,45].
- The child’s age and oral-motor development, ensuring readiness and safety for the introduction of new textures and foods (see Section 5.2).
- The absence of active Eosinophilic Esophagitis (EoE) or Food Protein Induced Enterocolitis Syndrome (FPIES) [41,46].

### 5.2. Age of Reintroduction—When?

Several scientific organizations, GPIFN and MAP, NICE, WAO DRACMA, and ESPGHAN, recommend maintaining a cow’s milk-free diet until 9–12 months of age and for at least 6 months (whichever is reached first) before starting the reintroduction of CMPs [3,4,28,30] (Table 4). Despite the overall consistency across the scientific societies’ statements regarding the recommended age and duration of the elimination diet before reintroduction, there are currently no data supporting these recommendations.

It is noteworthy that Ball et al. and d’Art et al. [20,27] reported the successful reintroduction of CMPs as early as 8 months of age. However, this approach was based on individualized risk assessment, conducted under appropriate clinical supervision, and initiated only when the child and caregivers were ready and confident to proceed. Parents needed to understand the concept of reintroduction, and recognize the importance of regular consumption of tolerated foods to sustain tolerance.

A younger age (<6 years) at the start of oral immunotherapy (OIT) appears to be associated with a higher likelihood of developing and maintaining tolerance, as well as fewer allergic reactions [44]. Moreover, evidence from milk OIT studies suggest that younger children and infants have a greater chance of achieving remission of CMPA through baked milk reintroduction [47].

### 5.3. Home Reintroduction Versus Hospital Oral Food Challenge—Where?

The decision whether baked milk reintroduction should be performed in hospital or at home is primarily based on individualized risk assessment [27,44]. Hospital-based reintroduction is recommended for children with a high-risk CMPA phenotype, including those with:

- A history of anaphylaxis, particularly to baked milk or at a very low threshold;
- Suspected or confirmed asthma or recurrent wheezing;
- High milk-specific IgE levels or large skin prick test (SPT) wheal size (>8 mm) [27,39,44].

An overview comparing hospital- and home-based approaches is provided in Table 5.

Initiating the milk ladder as an oral food challenge (OFC) in hospital ensures safety, reduces caregiver anxiety, and allows for objective symptom monitoring. In this controlled environment, children have immediate access to resuscitation equipment, emergency medications (e.g., intramuscular adrenaline, corticosteroids), and trained personnel capable of managing anaphylaxis and other severe reactions [27,39]. Standardized observation and documentation, consistent with PRACTALL guidelines, facilitate the reliable assessment and interpretation of symptoms [46]. Hospital-based OFC also enables the identification of cofactors, such as infections, asthma exacerbations, physical exertion, or non-steroidal anti-inflammatory drug (NSAID) use, that may influence allergic reactivity. In such cases, exposure should be postponed to minimize false-positive reactions and unnecessary dietary restrictions [15,25,40,48].

In contrast, the success of home-based reintroduction depends on caregiver competence, availability of emergency medication, and adherence to an individualized action plan [27]. Caregivers must be confident, trained to recognize early symptoms of allergic reactions (especially anaphylaxis), and have prompt access to emergency care. Home reintroduction is generally safe and more convenient, as most reactions are mild and manageable with prescribed rescue medications (e.g., antihistamines, adrenaline auto-injector). Its main limitation lies in the difficulty of identifying cofactors, which may lead to misinterpretation of mild or unrelated symptoms as allergic reactions [15,20,25].

In summary, children with high-risk CMPA should begin the milk ladder under hospital supervision, particularly those with a history of anaphylaxis, severe or uncontrolled asthma, very low reaction thresholds, or markedly elevated milk-specific IgE or SPT results, due to the increased risk of severe reactions and the need for emergency support [15,25,40,48]. The subsequent steps are usually also performed in a hospital setting; however, the final decision depends on the individual risk assessment, the level of parental anxiety, and shared decision-making with the parents. For children with low-risk IgE-mediated CMPA, home reintroduction of baked milk may be considered, provided that families are well trained and supported by qualified healthcare professionals [15,23,27].

## 6. Implementation of the Milk Ladder—Barriers and Facilitators

### 6.1. Food Aversion

In clinical practice, children often show aversion to specific foods, particularly those previously eliminated or associated with allergic reactions [49]. Such aversion may involve the food’s taste, texture, or smell, leading to delayed allergen reintroduction, difficulty completing the milk ladder, or temporary withdrawal from the protocol. These challenges are more common in children with coexisting neurodevelopmental disorders, including autism spectrum disorder and attention deficit hyperactivity disorder [50]. A case series by Wiszniewska et al., although in a small sample-size, demonstrated that previously acquired tolerance may be lost due to irregular or absent exposure to tolerated foods containing cow’s milk proteins [25]. Therefore, early identification of children with feeding difficulties, sensory hypersensitivity, or Avoidant/Restrictive Food Intake Disorder (ARFID) is essential, ideally before initiating the milk ladder protocol.

Recent systematic reviews emphasize that behavioral aspects of food allergy should be incorporated into clinical management. Food-related anxiety and aversion in allergic children may persist without targeted intervention [51]. Early involvement of psychologists or behavioral specialists, particularly for children showing strong food avoidance, can improve acceptance of milk-containing foods and adherence to the milk ladder protocol [51,52,53].

Before performing the OFC, parents should be asked about the types of foods their child accepts and discuss suitable forms of baked-milk-containing foods. Consultation with an experienced dietitian regarding recipe modification, especially for taste and texture, may further support children with food aversion [16,54].

### 6.2. Parental Anxiety

Food allergy-specific anxiety is a distinct phenomenon associated with increased distress, hypervigilance, and reduced quality of life among caregivers of children with food allergy [53]. In IgE-mediated CMPA, particularly in cases with a history of anaphylaxis, parental anxiety can be significant and may hinder progression through the milk ladder [20,48,49]. Such fear may also be transmitted to the child, reinforcing food avoidance and maladaptive feeding behaviors [20,48,49]. Excessive parental anxiety can result in unnecessary dietary restriction and reluctance to follow physician-recommended reintroduction plans. Providing psychological support and structured reassurance has been shown to reduce fear, improve adherence, and enhance family functioning [51].

### 6.3. Lack of Standardized Milk Ladder Protocol

The main challenges associated with the lack of milk ladder standardization are [16,19,41,55,56,57,58,59,60]:

- ***Variability in recipe composition and allergen content***
  The recipes used in the available milk ladders (Table 3) differ considerably in ingredient composition and proportions. The total CMP content per step also varies between protocols, making it difficult to compare clinical outcomes or define universal tolerance thresholds.
- ***Differences in heat processing and matrix effects***
  Variations in processing time and temperature, flour type (wheat vs. gluten-free), fat content, and moisture influence the degree of milk protein denaturation and matrix effects, thereby substantially altering the allergenicity of tested product.
- ***Inconsistent portion sizes and CMP content***
  Many protocols do not specify the exact amount of cow’s milk protein per serving (e.g., mg of CMP per step). The absence of analytical verification of CMP content limits clinical safety assurance and hinders consistent interpretation of outcomes, particularly in children with IgE-mediated CMPA.
- ***Lack of standardized criteria for continuation/discontinuation of milk ladder***
  Progression through the milk ladder may be based on fixed time intervals (e.g., 3–7 days per step) or on tolerance criteria. The absence of unified success parameters contributes to significant variability in clinical practice.
- ***Inconsistent patient selection criteria and safety thresholds***
  No consensus exists on criteria defining high-risk CMPA phenotypes (e.g., age < 6 years, specific IgE levels, SPT wheal size, or anaphylaxis history). This gap leads to heterogeneity in efficacy and safety outcomes. Some centers limit milk ladder use to low-risk children, whereas others initiate it in high-risk groups under close supervision.
- ***Limited reproducibility across studies and settings***
  Inconsistencies in protocols, study design, patient selection, and outcome reporting impede comparison and meta-analytical synthesis. Consequently, the available evidence remains insufficient to support high-certainty clinical recommendations.
- ***Lack of validated biomarkers of tolerance progression***
  Current milk ladder protocols lack standardized immunological markers (e.g., specific IgE, IgG4, basophil activation tests) that could help predict a child’s clinical response to various forms of heated milk-containing foods.

## 7. Impact of Food Composition and Processing on Milk Allergenicity

### 7.1. Milk-Containing Baked Products

The specific composition of baked products plays a key role in modulating the allergenic potential of milk proteins during thermal processing [56,57,61] (Table 6).

Wheat flour, commonly used in standard baked milk challenge recipes, is considered optimal for forming the food matrix that reduces CMPs allergenicity [56,57].

Gluten and starch create a viscoelastic network that promotes protein–protein and protein–carbohydrate interactions, leading to extensive denaturation of conformational epitopes, particularly β-lactoglobulin, with IgE reactivity reduced by over 95–99%. In contrast, heat-stable caseins undergo only partial degradation, with allergenicity reduced by approximately 30–40% [57], which may remain clinically relevant in patients with high casein-specific IgE.

Replacing wheat flour with gluten-free alternatives (e.g., rice, corn, oat, or barley flour) alters the matrix structure and thermal behavior [58]. The absence of gluten reduces matrix stability and may limit protein denaturation. Oat and barley flours, rich in β-glucans and amylose, modify viscosity and hydration, although their effects on allergenicity are not fully understood [57,58].

Adding fruits can affect allergenicity through pH changes, increased moisture, and polyphenol content. Acidic fruits promote protein unfolding, and polyphenols may bind milk proteins and reduce IgE accessibility, while excessive moisture can limit denaturation [56,61].

Other influential factors include baking time and temperature—longer or higher conditions enhance denaturation—as well as ingredient ratios and fat content. Lipids may stabilize milk proteins through hydrophobic interactions, decreasing thermal unfolding [61].

### 7.2. Fermented Products

The bacterial fermentation process used in yogurt production, involving *Lactobacillus bulgaricus*, *Streptococcus thermophilus*, and other lactic acid bacteria, reduces milk protein allergenicity primarily through proteolytic degradation of conformational and linear IgE-binding epitopes on major allergens such as β-lactoglobulin and α-lactalbumin [55]. Enzymatic hydrolysis generates smaller peptides with reduced IgE reactivity, which is shown to significantly decrease antigenicity and IgE-binding capacity in both in vitro and in vivo studies [55].

The degree of allergenicity reduction depends on the bacterial strains and their proteolytic activity. For instance, *Lactobacillus delbrueckii* subsp. *bulgaricus* degrades key β-lactoglobulin epitopes, leading to up to a 35% reduction in IgE reactivity, while co-fermentation with other lactic acid bacteria may further enhance this effect [59]. Additionally, metabolites produced during fermentation (e.g., small organic acids and bioactive peptides) contribute to structural modifications of milk proteins, further decreasing their allergenic potential [55].

## 8. Shaping the Future of Milk Ladders: Standardization and Innovation

The future of milk ladders in CMPA lies in improving standardization, safety, and clinical efficacy to enable their potential inclusion in international allergy management guidelines [16].

### 8.1. Commercially Available Foods

Achieving global harmonization requires the development of protocols based on quantified CMP content and consistent definitions of allergen doses at each stage. Recent initiatives have therefore emphasized the use of standardized, commercially available products (e.g., biscuits or muffins) in the initial steps of the milk ladder [62]. These ready-to-use products can substantially improve accuracy, reproducibility, and safety by minimizing variability associated with home preparation, thereby facilitating broader clinical implementation worldwide [62].

### 8.2. Omalizumab

An emerging approach in CMPA management involves the integration of immunomodulatory therapies, particularly omalizumab, a monoclonal anti-IgE antibody [19,63]. Omalizumab was recently approved by the U.S. Food and Drug Administration (FDA) as an adjunctive treatment for food allergies, reflecting growing evidence of its role in facilitating safer and more effective desensitization [19]. Clinical trials have demonstrated that omalizumab can accelerate tolerance acquisition, reduce the frequency and severity of allergic reactions, and improve quality of life in patients with severe or persistent allergic phenotypes [63].

According to the 2022 WAO consensus statement on biologics in food allergy management, omalizumab may be considered as an adjunct to OIT or structured reintroduction protocols, such as the milk ladder, in patients at high risk of anaphylaxis or with previous OIT failure. Its use has been shown to enhance safety, shorten up-dosing phases, and enable desensitization in children otherwise unable to tolerate standard protocols. Further research is warranted to define optimal treatment duration, cost-effectiveness, and long-term outcomes when integrated into CMPA desensitization strategies [64].

### 8.3. Low-Dose Early Exposure

Another promising strategy involves early, hospital-supervised exposure to a low dose of milk before ladder initiation. This approach has been shown to accelerate progression primarily by reducing parental anxiety and improving caregiver confidence in continuing the protocol at home [20].

In a five-year follow-up study by Corcoran et al. (2025) [65], infants who received a single low-dose exposure equivalent to the eliciting dose ED05 at diagnosis achieved tolerance to cow’s milk significantly earlier than those who followed standard ladder-based care. Although final tolerance rates at five years were similar between the intervention and control groups (90% vs. 76%, *p* = 0.21), the timing of tolerance acquisition was notably accelerated in those exposed to the ED05. These findings support the hypothesis that early, controlled exposure may promote faster immune modulation and desensitization while maintaining safety under medical supervision [65].

### 8.4. Milk Ladder Based on Measured Levels of CMPs in Foods

A recent study by Simmons et al. (2025) quantified the levels of two major milk allergens, β-lactoglobulin (Bos d5) and β-casein (Bos d11), in foods commonly used in the iMAP milk ladder using ELISA [66]. Cookies, muffins, pancakes, cheese, yogurt, and milk were analyzed to assess the influence of the food matrix, heating conditions, and industrial milk processing on detectable allergen levels.

The study demonstrated that the order of foods along the milk ladder varied depending on whether it was based on calculated total milk protein compared with the measured Bos d5 or Bos d11 [66]. For instance, pancakes, despite the higher cow’s milk content per portion, showed lower Bos d5 levels than muffins, likely due to a higher heating temperature and thinner matrix structure. Conversely, Bos d11 levels did not follow a consistent increase across the ladder, reflecting the greater heat stability of casein compared with whey proteins. Notably, detectable allergen levels were also affected by milk processing, with Bos d5 significantly reduced in ultra-high temperature (UHT) milk compared with raw or pasteurized milk.

These findings provide novel evidence that food matrix composition, heating conditions, and protein type critically influence allergen detectability and, consequently, the immunological profile of each ladder step. From a clinical perspective, this suggests that the child’s sensitization pattern (e.g., Bos d5- vs. Bos d11-dominant) might, in the future, guide individualized ladder progression. However, this concept requires further validation in clinical trials before routine implementation.

## 9. Conclusions

The milk ladder provides a structured, stepwise framework for the gradual reintroduction of CMPs in children with CMPA, balancing safety with the goal of inducing tolerance. Although, it was primarily developed for only children with mild-to-moderate non-IgE-mediated CMPA, new evidence shows that it may be also safely applicable to selected patients with IgE-mediated CMPA. While in patients with a high-risk phenotype (defined as a history of anaphylaxis, suspected or confirmed asthma or recurrent wheezing, and/or high milk-specific IgE levels or a large SPT wheal size), the reintroduction of CMPs may be more challenging, this review provides a summary of evidence and practical insights on the implementation of the milk ladder.

Standardization of food composition, portion size, and allergen quantification remains essential to improving reproducibility and comparability across studies. Recent advances, including the use of commercially available standardized foods, quantified allergen levels, and adjunctive immunomodulatory therapies such as omalizumab, offer promising directions for optimizing clinical outcomes. Early, low-dose, supervised exposure and the integration of behavioral and psychological support further enhance the success and safety of milk reintroduction.

Continued research is required to establish evidence-based, personalized protocols that balance safety, efficacy, and practicality in routine allergy management. Further studies are needed to identify biomarkers that could predict clinical responses to baked milk. Moreover, the optimal time of the reintroduction of cow’s milk proteins and the appropriate interval between subsequent steps still need to be determined.

## Figures and Tables

**Table 2:** Summary of recommendations regarding the milk ladder’s use for the reintroduction of cow’s milk proteins in children with CMPA.

Organization	Statements
**GPIFN and MAP 2019** [30]	A planned reintroduction or supervised challenge is then needed to determine if tolerance has been acquired. Reintroduction at home using milk ladder is recommended in children with mild to moderate non-IgE-mediated CMPA without history at any time of immediate onset symptoms (in case of current atopic dermatitis, with negative serum specific IgE or SPT to cow’s milk). The iMAP Home Milk Ladder should not be used in other presentations such as IgE-mediated CMA or severe non-IgE-mediated CMA (e.g., FPIES).
**NICE 2019** [28]	In children with mild-to-moderate non-IgE-mediated CMPA tolerance to cow’s milk protein should be assessed using a ‘milk ladder’ and monitored for the return of symptoms. In children with IgE-mediated CMPA, the follow-up may include serial allergy testing and subsequent OFC to test for acquired tolerance.
**WAO DRACMA 2023** [4]	The milk-ladder can be used for reintroduction in non-IgE mediated food allergy (FPIAP, and food protein induced enteropathy—FPE) and might be considered in carefully selected cases of IgE-mediated CMPA and CM-FPIES to evaluate for tolerance after a period of therapeutic elimination diet. In IgE mediated CMPA, reintroduction to establish tolerance should be guided by the severity of symptoms and specific IgE and/or skin prick test. If there are still detectable levels of specific IgE, reintroduction has to be performed under physician supervision in a medical settings, especially if the initial symptoms were severe.
**ESPGHAN 2024** [3]	The OFC after first period of therapeutic diet can be performed in a similar fashion to that after the diagnostic elimination diet or according to the milk ladder, starting with small amounts of baked milk. Home introduction protocols are safe in non-IgE-mediated CMPA. Specific IgE to cow’s milk should be measured before the OFC and guide timing of the OFC. Emphasizes that standardisation of the home challenge applying milk ladder adapted to local dietary habits is recommended.
**EAACI 2024** [31,32]	In the non-IgE-mediated cow’s milk-allergic breastfed infant that is on solids, a milk ladder may be used (not including FPIES), but there are no data on its efficacy. In patients with confirmed IgE-mediated food allergy, avoidance of the food (or form of the food) to which the patient is allergic is recommended. Dietary management should support expansion of the diet when clinically indicated, e.g., baked milk and/or egg.

GPIFN—General Practitioners Infant Feeding Network, EAACI—European Academy of Allergy and Clinical Immunology, ESPGHAN—European Society for Paediatric Gastroenterology, Hepatology and Nutrition, FPE—food protein-induced enteropathy, FPIAP—food protein-induced allergic proctocolitis, FPIES—food protein-induced enterocolitis syndrome, IgE—immunoglobulin E, MAP—Milk Allergy in Primary Care, NICE—National Institute for Health and Care Excellence, SPT—skin prick test, WAO DRACMA—World Allergy Organization: Diagnosis and Rationale for Action against Cow’s Milk Allergy.

**Table 3:** Comparison of MAP/iMAP Milk Ladders and National Milk Ladder Protocols.

Milk Ladder Protocol (Country)	Year	No of Steps	Reintroduced Foods	Foods Number per Step	Allergen Dose Specified (Initial → Final)
***MAP and iMAP Milk Ladders***					
**MAP (UK)** [17]	2013	12	Malted milk biscuits, Garibaldi biscuits/digestives, muffins/cupcakes, pancakes, Shepherd’s pie, lasagne, mini pizza, milk chocolate, yoghurt, cheese, sterilized milk, pasteurized milk	1	Yes, 95 mg → 7.2 g
**iMAP (UK)** [14]	2017	6	Cookies/biscuits, muffins, pancakes, cheese, yoghurt, pasteurized milk	1	Yes, 35 mg → 6.9 g
***National Adaptation of the MAP/iMAP Milk Ladders***					
**BSACI (UK)** [35]	2014	4	Biscuits, cakes, muffin, waffles, scotch pancakes, butter, margarine, cheese powder flavouring, custard, cheese sauce, pizza, rice pudding, chocolate, chocolate coated items, fermented desserts, yogurt, fromage frais, ice cream, mousse, pasteurised milk	Multiple	No
**Canadian Milk Ladder (Canada)** [37]	2021	4	Muffins/cupcakes, cookies, pancakes/crepes, waffles, pizza, boiled milk, cheese, yoghurt, ice cream, milk	2–4	No
**Indian Milk Ladder (India)** [33]	2023	6	Marie biscuit, Maida Diamond Biscuit, milk cookies, halwa, ragi sari/kanji with milk, ragi dosa, rava idli, gulab jamun (dry), rasagullah, rice Kheer, paneer, shrikand or yoghurt, milk	2–4	Yes, 50 mg → 8.68 g
**Mediterranean Milk Ladder (Greece)** [38]	2023	7	Beef burgers, oat cookies with olive oil, sweet whole wheat muffins with berries or raisins, whole wheat savoury muffins, mediterranean type pureed potato, crepes with whole wheat flour, bread with cheese, olives, and tomato, lentil burger with cheese, rice pudding, bechamel, yogurt, cocoa-banana ice-cream, avocado-cocoa mousse	1	Yes, 70 mg → 3.2 g
**Spanish (Spain)** [36]	2023	4	Cookies, muffins, sweet pancakes, salted pancakes, croquettes, bechamel, Spanish omelette, banana puree, fillings for stew, French omelette, yoghurt, milk	1–6	Yes, 95 mg → 6.2 g
**German (Germany)** [29]	2023	6	Pastry, rusk, muffin, pancake, pizza, rice pudding, cheese, yoghurt, milk	1–3	Yes, 8 mg → 7 g
**Turkish (Turkey)** [39]	2024	4	Biscotti twice baked cake, baked cake variations (muffins, biscuits), pancake, crepe, waffle, yoghurt in soup (Yayla soup), Tarhana soup, pizza, yoghurt, cheese (curd cheese, quark cheese, labneh cheese), pudding, mousse, ice cream, whipped cream, milk	1–3	Yes, 85 mg → not reported

BSACI—British Society for Allergy & Clinical Immunology; MAP—Milk Allergy in Primary Care; iMAP—international Milk Allergy in Primary Care.

**Table 4:** Summary of recommendations on age of cow’s milk re-challenge to test for acquired tolerance.

Organization	Time for Cow’s Milk Re-Challenge to Test for Acquired Tolerance
NICE 2019 [28]	≥6 mo. of elimination diet or at 9–12 month of age
GPIFN and MAP 2019 [30]	≥6 mo. of elimination diet or at 9–12 month of age (whichever is reached first)
WAO DRACMA 2023 [4]	≥6 mo. of elimination diet or up to 9–12 month of age (whichever is reached first)
ESPGHAN 2024 [3]	≥6 mo. of elimination diet or at 12 month of age

ESPGHAN—European Society for Paediatric Gastroenterology, Hepatology and Nutrition, GPIFN—General Practice Infant Feeding Network, MAP—Milk Allergy in Primary Care guideline, NICE—National Institute for Health and Care Excellence, WAO DRACMA—World Allergy Organization: Diagnosis and Rationale for Action against Cow’s Milk Allergy.

**Table 5:** Comparison of Hospital-Based Oral Food Challenge versus Home-Based Reintroduction [12,15,16,19,20,27,28,39,46,48].

Aspect	Hospital-Based Oral Food Challenge	Home-Based Reintroduction
**Supervision**	- Under direct medical supervision by trained personnel in a controlled setting.	- Reintroduction conducted by appropriately trained caregivers, with remote or follow-up medical supervision.
**Emergency** **Support**	- Immediate access to resuscitation equipment and emergency medication (e.g., adrenaline, corticosteroids) administered by qualified personnel.	- Prescription of rescue medication (e.g., adrenaline auto-injector), providing adequate training to caregivers if required, confirming access to emergency care if needed.
**Monitoring and Documentation**	- Standardized, objective assessment in line with the PRACTALL guidelines, with structured observation and symptom scoring.	- Based on subjective parental observation; documentation may be subjective and less consistent.
**Cofactors** **Management**	- Identification of potential cofactors (infection, asthma exacerbation) and decision on temporary deferral, if needed; decreased risk of false-positive reactions.	- Difficulties with controlling and identifying potential cofactors that may affect interpretation of allergic reaction.
**Patient Selection**	- Recommended for children with a high-risk phenotype of CMPA (e.g., prior anaphylaxis, uncontrolled asthma, high level of milk-specific IgE or wheal size in SPT).	- Recommended for children with a mild-to-moderate-risk phenotype of CMPA based on an individualized risk assessment.
**Advantages**	- Ensure safety, objective symptoms interpretation, immediate intervention, reduced parental anxiety, standardized procedures.	- Convenient for children and caregivers, low resource requirements, promotes gradual exposure and family involvement.
**Limitations**	- Limited availability, higher cost, delayed reintroduction due to long waiting lists.	- Variable caregiver competence and adherence to protocol, unequal access to emergency medication, risk of uncontrolled cofactors.

**Table 6:** Key Muffin Ingredients and their Potential Impact on Milk Protein Allergenicity [56,57,61].

Ingredient	Mechanistic Effect	Presumable Impact on CMPs Allergenicity	Comments
**Wheat flour**	Forms gluten–starch matrix during baking	Major reduction in β-lactoglobulin allergenicity (>95%)	Preferred matrix in baked milk challenges
**Gluten-free flours**	Lack of gluten, less stable matrix	Less protein denaturation; higher residual allergenicity	Outcomes are less predictable; children tolerating muffins with wheat flour may react to muffins with gluten-free flour
**Fruits**	Lower pH and introduce polyphenols	May reduce IgE binding but excess moisture can limit denaturation	Effect depends on fruit type
**Sugars**	Participate in Maillard reactions	Usually reduce IgE binding but may create neoepitopes	Controlled sugar levels are important
**Fats**	Stabilize proteins via hydrophobic interactions	Can preserve allergenic epitopes	Standardize fat content for reproducibility

## Abbreviations

The following abbreviations are used in this manuscript:

ARFID	Avoidant/Restrictive Food Intake Disorder
BM	baked milk
CI	confidence interval
CMPA	cow’s milk protein allergy
CMPs	cow’s milk proteins
DRACMA	Diagnosis and Rationale for Action against Cow’s Milk Allergy
EAACI	European Academy of Allergy and Clinical Immunology
ELISA	enzyme-linked immunosorbent assay
EoE	eosinophilic esophagitis
ESPGHAN	European Society for Paediatric Gastroenterology, Hepatology and Nutrition
FDA	Food and Drug Administration
FPE	food protein-induced enteropathy
FPIAP	food protein-induced allergic proctocolitis
FPIES	food protein-induced enterocolitis syndrome
GPIFN	General Practitioners Infant Feeding Network
IFAN	Irish Food Allergy Network
iMAP	international Milk Allergy in Primary Care
IgE	immunoglobulin E
MAFM	more allergic form of milk
MAP	Milk Allergy in Primary Care
ML	milk ladder
NICE	National Institute for Health and Care Excellence
NSAID	non-steroidal anti-inflammatory drug
OFC	oral food challenge
OIT	oral immunotherapy
OR	odds ratio
PRACTALL	practical allergy consensus (joint EAACI/AAAAI recommendations)
RCT	randomized controlled trial
sIgE	specific immunoglobulin E
SPT	skin prick test
UHT	ultra-high temperature
WAO	World Allergy Organization
